# Phenotypes in siblings with homozygous mutations of *TRAPPC9* and/or *MCPH1* support a bifunctional model of MCPH1

**DOI:** 10.1002/mgg3.400

**Published:** 2018-04-24

**Authors:** Sarah Duerinckx, Marije Meuwissen, Camille Perazzolo, Laurence Desmyter, Isabelle Pirson, Marc Abramowicz

**Affiliations:** ^1^ Department of Medical Genetics Hôpital Erasme and IRIBHM Université Libre de Bruxelles Brussels Belgium; ^2^ Department of Medical Genetics Antwerp University Hospital Antwerp Belgium

**Keywords:** centrosome, consanguinity, DNA damage repair, exome, microcephaly

## Abstract

**Background:**

Autosomal recessive intellectual disability (ARID) is vastly heterogeneous. Truncating mutations of *TRAPPC9* were reported in 8 ARID families. Autosomal recessive primary microcephaly (MCPH) represents another subgroup of ARID, itself very heterogeneous, where the size of the brain is very small since birth. *MCPH1* plays a role at the centrosome via a BRCT1 domain, and in DNA Damage Repair (DDR) via BRCT2 and BRCT3, and it is not clear which of these two mechanisms causes MCPH in man.

**Methods:**

We studied the phenotype and sequenced the exome in two siblings with MCPH and their unaffected sister.

**Results:**

Homozygous mutations of *TRAPPC9* (p.Leu178Pro) and of *MCPH1* (p.Arg741X) were found in both affected siblings. Brain MRI showed anomalies previously associated with TRAPPC9 defects, supporting the implication of *TRAPPC9* in the phenotype. Importantly, the asymptomatic sister with normal head size was homozygous for the *MCPH1* truncating mutation and heterozygous for the *TRAPPC9* mutation.

**Conclusion:**

The affected siblings represent the first ARID cases with a *TRAPPC9* missense mutation and with microcephaly of prenatal onset of. Furthermore, their unaffected sister represents strong evidence that the lack of MCPH1 BRCT3 domain does not cause MCPH in man, supporting a bifunctional model of MCPH1 where the centrosomal function is involved in brain volumic development and not the DDR function.

## 1 INTRODUCTION

Autosomal recessive intellectual deficiencies (ARID) are a very heterogeneous subgroup of intellectual deficiencies (Najmabadi et al., 2011). *TRAPPC9* mutations and *MCPH1* mutations each cause a type of ARID.

TRAPPC9 deficiency was identified as a cause of ARID in eight families worldwide (Khattak & Mir, 2014). Additional features are microcephaly of postnatal onset, speech delay, and abnormalities of the corpus callosum, cerebellum, and white matter (Kakar et al., 2012). TRAPPC9 is part of the Trafficking Protein Particle (TRAPP) Complex II, and is involved in intra‐Golgi and endosomal trafficking via interactions with TRAPPC2 and TRAPPC10 (Zong et al., 2012). TRAPPC9 was also shown to play a role in nuclear factor kappaB (NF‐kB) activation (Philippe et al., 2009).

Contrary to microcephaly of postnatal onset (i.e., secondary microcephaly), primary microcephaly results from insufficient production of mature neurons during neurogenesis, and presents with a congenitally small brain (Passemard, Kaindl, & Verloes, 2013). *M*icro*C*ephaly *P*rimary *H*ereditary (MCPH) refer to a group of nonsyndromic, autosomal recessive primary microcephalies. MCPH is largely heterogeneous, with at least 16 different causal genes.

Microcephalin (*MCPH1*) is one of the first genes associated with MCPH. MCPH1 is expressed at the centrosome and plays roles in mitotic spindle alignment and mitotic cycle checkpoints. MCPH1 deficiency uncouples mitosis and the centrosomal cycle, causing premature mitotic entry, with premature neurogenic production and depletion of neural progenitors (Pulvers, Journiac, Arai, & Nardelli, 2015). Interestingly, MCPH1 also plays a role in DNA damage repair (DDR). MCPH1 contains three BRCT domains that were predicted to be crucial for MCPH1 function (Pulvers et al., 2015). N‐terminal BRCT1 is necessary for centrosomal localization in chicken cells, whereas C‐terminal BRCT2 and BRCT3 are required for ionizing radiation‐induced nuclear foci (IRIF) formation (Pulvers et al., 2015). BRCT2 and BRCT3 bind E2F1 to form a complex that transactivates *BRCA1* and *CHK1* (Yang, Lin, & Lin, 2008). BRCT2 and BRCT3 also interact with Cdc27, a subunit of the anaphase‐promoting complex (Singh, Wiltshire, Thompson, Mer, & Couch, 2012) and H2AX (Singh et al., 2012). Other DDR genes are also associated with primary microcephaly, for example, *BLM* or *LIG4* (Maciejczyk et al. (2016), Berg et al. (2011)), so the exact mechanism by which *MCPH1* mutations cause microcephaly, either centrosomal or via DDR, is not clear.

We here report on two siblings with severe ID, microcephaly and hypoplasia of the corpus callosum, a homozygous *TRAPPC9* mutation, and a homozygous *MCPH1* truncation of BRCT3, and their normal sister with the homozygous *MCPH1* mutation only.

## 2 MATERIAL AND METHODS

The two affected siblings, a boy and a girl, and their nonaffected sister, were born to Moroccan parents originating from the same small village, and likely consanguineous.

The affected son was referred at 16 years of age for psychomotor delay and severe ID, microcephaly and hyperkinesia. The HC was 52 cm (−2.8SD), height was 161 cm and weight 61.6 kg. He had sphincter control and could express himself with a few words. Brain MRI showed hypoplasia of the corpus callosum and mild colpocephaly. Head circumference (HC) at birth was not known.

A younger sister was referred at 10 years of age for psychomotor delay and severe ID, microcephaly, hyperkinesia, and epilepsy. HC was <P3, height at P10, and weight at P25. She was not toilet‐trained, not able to speak, avoided ocular contact, and walked with an enlarged basis of sustentation. Her head was reportedly small at birth. At 13 years, her HC of 48.5 cm (−4SD). MRI showed atrophy of the corpus callosum and cerebellum, and some abnormal signals in the supratentorial white matter. Fundi were normal with intermittent strabismus. A metabolic work‐up, standard karyotype, and FISH analysis of all subtelomeric regions were normal.

HC and intellect of the parents and nonaffected sister were strictly normal: 59 cm in father, 57 cm in mother, and 57 cm in nonaffected sister whose height was 160 cm.

SNP genotyping revealed a large homozygous region of 14 Mb at 8q24 encompassing *TRAPPC9*, and a small homozygous region of 0.4 Mb in 8p23 encompassing *MCPH1*.

For whole‐exome sequencing, the two probands’ genomic DNAs were sheared and exonic sequences enriched using Roche SeqCap EZ Human Exome v3.0 (64 Mb) DNA capture. Sequencing was performed in the affected boy by AROS applied biotechnology (http://arosab.com/), and in the affected girl on a HiSeq1500 Illumina sequencer at the BRIGHTcore BRussels Interuniversity Genomics High Throughput core (http://www.ngs.brightcore.brussels/). Raw sequences were aligned to the reference genome GRCh37 using BWA algorithm version 0.7.10 (Li and Durbin (2009)), duplicated reads were then marked using Picard version 1.97 (http://broadinstitute.github.io/picard/), alignment quality was improved using the GATK (DePristo et al. (2011)) realigner and base recalibrator version 2.7, and finally, variants were called using GATK Haplotype Caller version 2.7. The resulting variant set was annotated and filtered using the Highlander software (http://sites.uclouvain.be/highlander/). Variants were filtered for quality criteria, allelic frequency <0.5%, functional impact, homozygous genotype, and cosegregation in the two affected sibs. Variants were then sorted by decreasing Combined Annotation Dependent Depletion (CADD) score (Kircher et al., 2014). The variants of interest were confirmed by Sanger sequencing. DNA was amplified using a standard Polymerase Chain Reaction (TRAPPC9 exon 2, forward primer: CTCCCAGGGTAGGCTCTCAG, reverse primer: AAGAGCCGGGAGTCATACAG; MCPH1 exon 13, forward primer: TTCGCCTACGCTATGGAGACT, reverse primer: ATCTGGACCACACCACAGCG). The PCR product was purified with Exosap‐IT (Affymetrix), and sequenced by the company Beckman Coulter Genomics.

All procedures complied with the ethical guidelines of Hôpital Erasme—Université Libre de Bruxelles, whose Ethics Committee approved our study protocol under reference P2005/076 (Ethics Committee Erasme Hospital, OMO21). Written informed consent to participate in our study was obtained from the patient's representative.

## 3 RESULTS

**Figure 1:**
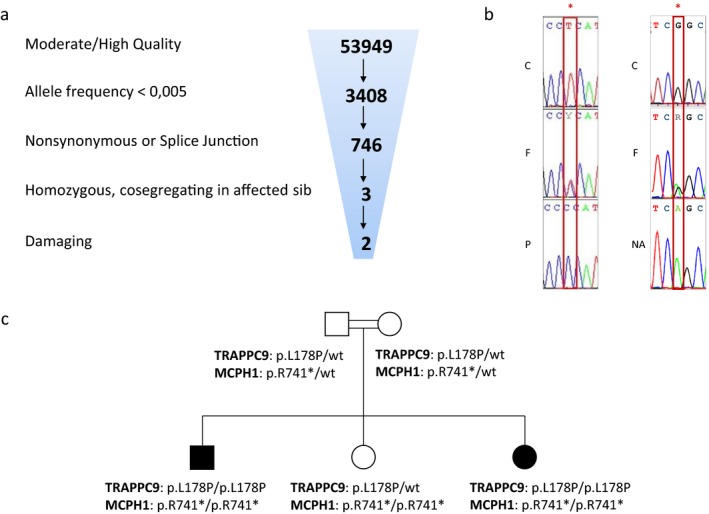
*TRAPPC9* and *MCPH1* mutations. (a) Exome sequencing data from one of the probands, showing the filtering parameters used to sort through the variant dataset. (b) Sanger sequencing of part of exon 2 of the *TRAPPC9* gene (left panel), and of part of exon 13 of the *MCPH1* gene (right panel). The *TRAPPC9* T to C mutation (*) at position 533 of the coding DNA sequence was found homozygous in the proband (P), and heterozygous in his father (F); a normal sequence is shown in an unrelated control subject (C). The *MCPH1* C to T mutation (*) at position 2221 of the coding DNA sequence was found homozygous in the nonaffected sib (NA), and heterozygous in their father (F); a normal sequence is shown in an unrelated control subject (C). (c) Family tree showing the consanguineous parents, the two probands and the nonaffected child, and their genotypes for *TRAPPC9* and *MCPH1* mutations

The exome sequencing data from the two affected children were filtered for rare (allele frequency <0.5%), nonsynonymous or splice‐junction variants that were homozygous in both affected siblings. Three such variants were identified in the *MCPH1* gene (c.2221C>T p.Arg741X), the *TRAPPC9* gene (c.533T>C p.Leu178Pro) and the *COL22A1* gene (c.1793G>A p.Arg598Gln) (Figure 1). The latter variant was predicted to be tolerated by several effect prediction programs and was thus not further considered.

**Figure 2:**
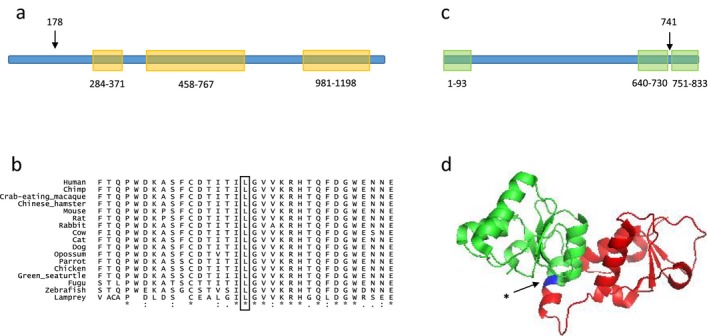
TRAPPC9 and MCPH1 conservation and domains. (a) Linear presentation of the TRAPPC9 protein, showing the three Trs120 domains (residues 284–371, 458–767, 981–1198). Arrow, position of the Leu178Pro mutation. (b) Alignment of TRAPPC9 amino acids sequence (residues 162–194) in multiple species (UCSC). “*” residue identical in all species. “:” conserved substitutions. “.” semiconserved substitutions. (c) Linear presentation of the MCPH1 protein, showing the three BRCT domains (residues 1–93, 640–730, 751–833). Arrow, position of the Arg741X mutation. (d) Crystal structure (PDB 3t1n Singh et al. (2012)) of MCPH1 tandem BRCT domains (residues 640–835). The portion of the protein truncated by the mutation appears on the right side of the arrow.

The c.2221C>T homozygous truncating mutation occurred in exon 13 of the 14‐exons *MCPH1* gene (transcript_ensembl ENST00000344683) changing the Arginine at position 741 of the polypeptide into a stop codon, p.Arg741X (Figure 1). This change, at chr8:6478981, was encompassed in a short 0.4 Mb homozygous stretch (chr8:6538837‐6906207). The variant frequency was 2.5 × 10^−5^ in the Exome Aggregation Consortium (Lek et al., 2016) with only 2 alleles reported, both heterozygous. The variant was absent from 1000G, GoNL, and our in‐house database. The mutation was located just upstream of the BRCT3 domain of the protein which spans amino acid residues 751 through 833, resulting in a truncation of this domain (Figure 2). Sanger sequencing confirmed homozygosity of the mutation in the two probands and heterozygosity in both parents. Importantly, Sanger sequencing also showed homozygosity of the *MCPH1* mutation in the nonaffected sister, who had a normal phenotype with a normal head size (Figure 1).

A nonsynonymous homozygous mutation was found in exon 2 of the 23‐exons *TRAPPC9* gene, c.533T>C (transcript_ensembl ENST00000389328) changing the Leucine at position 178 of the polypeptide into a Proline, p.Leu178Pro (Figure 1). This change, at chr8:141461234 (GRCh37), was encompassed by a 14 Mb homozygous stretch (chr8:128066556‐142227859), consistent with homozygosity by descent (autozygosity) for this chromosomal segment. The variant was not present in the public databases ExAC, 1000G, GoNL, nor in our in‐house database. The variant was predicted to be deleterious by 5 different programs (damaging in SIFT, probably_damaging in pph2, deleterious in LRT, disease_causing in mutation_taster, medium in mutation_assessor), and had a CADD score of 22. Leu178 is very conserved in vertebrates; UCSC Multiz Alignments of 100 Vertebrates (https://genome.ucsc.edu/) showed the presence of a Leucine in all species at this position (Figure 2). The mutation was located in the TRAPP II complex Trs120 domain of the TRAPPC9 protein which spans amino acid residues 99 to 1206. *TRAPPC9* is predicted to be intolerant to variation by ExAC (Lek et al., 2016; *z* score = 1.64) and RVIS programs (percentile 2.77%, meaning that only 2.77% of all the genes are more intolerant to variation than *TRAPPC9*) (not shown). In particular, exon 2 as well as the Trs120 domain of *TRAPPC9* gene were predicted to be extremely intolerant to variation by the subRVIS program (percentiles being, respectively, 1.556% and 0.066%) (exon 2 Supplementary File S1; Trs120 domain not shown) (Gussow, Petrovski, Wang, Allen, & Goldstein, 2016). Sanger sequencing confirmed homozygosity of the mutation in the two probands and heterozygosity in both parents as well as in the nonaffected sister (Figure 1).

## 4 DISCUSSION

We report on two siblings with microcephaly and severe ID in whom exome sequencing revealed homozygous mutations in two genes, *TRAPPC9* and *MCPH1*.

The patients’ phenotype was consistent with a TRAPPC9 defect, which is characterized by severe intellectual deficiency (ID), postnatal microcephaly, abnormalities of the corpus callosum, cerebellum, and white matter, as found in our patients. Together with the intolerance to variation in *TRAPPC9* exon 2; the absolute conservation of Leu178 in vertebrates; and the predicted pathogenicity of the p.Leu178Pro protein change, consistency of the phenotype provides strong evidence for causality of this genetic variant. To date, all reported *TRAPPC9* mutations were nonsense and splice variants (Supplementary File S2), and this is to our knowledge the first missense mutation of *TRAPPC9*. It is also the first case with congenital microcephaly (see below).

TRAPPC9 is expressed in neurons, and *TRAPPC9* mutations could impair neuronal development via defective vesicular trafficking. Alternatively, or perhaps in addition, the mechanism might involve nuclear factor kappaB (NF‐kB) activation. A defective activation of the NF‐kB pathway has been reported in skin fibroblasts from a patient carrying a biallelic *TRAPPC9* mutation (Philippe et al., 2009). According to ELM (Dinkel et al. (2016)), Leu178 is part of a kinase interaction motif, so this region may have a role in interaction with IKBKB or NIK and activation of the NF‐kB pathway.

*MCPH1* is composed of 14 exons, and all human mutations reported to date were found in exons 1 to 11, consisting of large deletions, small insertions or deletions, nonsense and missense mutations (Pulvers et al., 2015). No disease‐causing variant has been reported in the last three exons.

Experiments on chicken cells using various truncated forms of chicken Mcph1 showed that BRCT1 is necessary for centrosomal localization, whereas BRCT2 and BRCT3 are required for IRIF formation and response to DNA damage (Pulvers et al., 2015). The study of the two major transcripts of MCPH1 (full‐length MCPH1, and a transcript lacking exons 9 to 14) also showed that the C‐terminus of the protein was necessary for DNA damage response. Indeed, the truncated isoform was able to complement the defective chromosome condensation in human MCPH1‐deficient cells, but was not able to form MCPH1 foci after irradiation (Gavvovidis et al., 2012). Similarly, in a mouse model lacking only the BRCT3 domain, body size and brain weight were normal, and the PCC phenotype was not present cytogenetically (Trimborn et al., 2010).

Taken together, these data suggest that deletion of BRCT3 does not cause human microcephaly. Homozygous truncation of this domain, or any biallelic truncating mutations, have never been reported in normal subjects, however. Interestingly, ExAC mentions one homozygous genotype for a *MCPH1* gene mutation, p.Glu521Ter, which is predicted to truncate BRCT2 and BRCT3. The phenotype, whether strictly normal or microcephalic, is not reported, however.

Here we show a biallelic truncation of the BRCT3 domain in the normal, unaffected sister. Our observation is consistent with the full‐length MCPH1 isoform not being required for brain volumic development, and hence that the mechanism causing MCPH consists of a centrosomal localization defect, and not a DNA damage response defect. It supports a bifunctional model of MCPH1 where the centrosomal function only is involved in brain volumic development.

Congenital, primary microcephaly in our patients suggests that *TRAPPC9*, like MCPH‐associated genes, plays a role in the production of the pool of neural progenitors that will allow for volumic development of the brain. We cannot rule out, however, that congenital microcephaly might have resulted from a combined effect of the *TRAPPC9* and the *MCPH1* mutations, either via a digenic interaction, or independently with the resulting phenotype being an overlap of features of both defects. The normal phenotype in the *MCPH1*‐mutated sister nevertheless strongly argues against the latter hypothesis.

## 5 CONCLUSION

Our observation shows that truncation of the third BRCT domain of MCPH1 is consistent with a normal phenotype, arguing strongly for the hypothesis that *MCPH1* mutations cause MCPH by a centrosomal defect rather than by a DNA damage response defect. We also describe the first ID‐associated missense mutation in *TRAPPC9*.

## CONFLICT OF INTEREST

The authors declare that they have no competing interests.
