# Supplementary material for: Phenotypes in siblings with homozygous mutations of TRAPPC9 and/or MCPH1 support a bifunctional model of MCPH1
Source: Mol Genet Genomic Med. 2018 Apr 24;6(4):660–5. doi: 10.1002/mgg3.400 (PMC6081227; doi:10.1002/mgg3.400)
Supplement: Supplementary file 1 [file MGG3-6-660-s001.pdf]

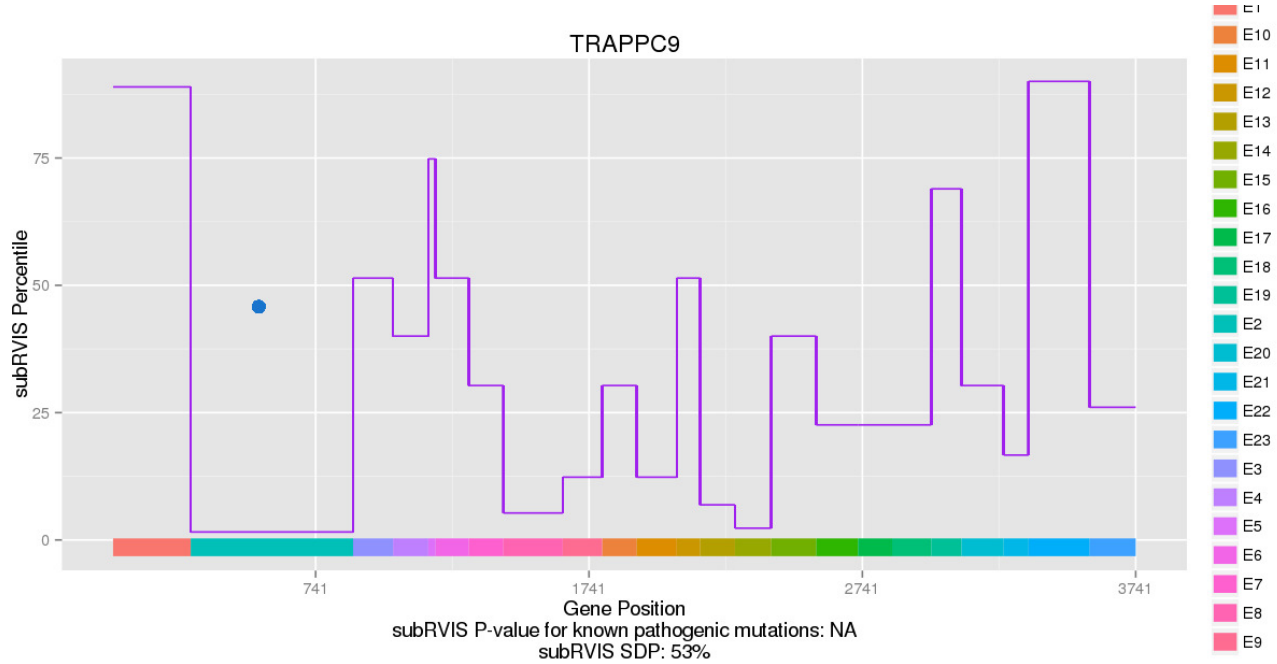

Supplementary File 1. **Tolerance to variation of TRAPPC9 exons.** SubRVIS percentiles of all TRAPPC9 exons. Lower subRVIS percentiles correspond to more intolerant regions. The blue dot shows the location of the residue Leu178. Exon 2 has a subRVIS score of 1,56, meaning that only 1,56 % of all the other exons from the genome have a more intolerant score. The high subRVIS SDP (53%) indicates a high degree of variation in the intolerance scores across the gene's exons.[17]
