# Supplementary material for: Phenotypes in siblings with homozygous mutations of TRAPPC9 and/or MCPH1 support a bifunctional model of MCPH1
Source: Mol Genet Genomic Med. 2018 Apr 24;6(4):660–5. doi: 10.1002/mgg3.400 (PMC6081227; doi:10.1002/mgg3.400)
Supplement: Supplementary file 2 [file MGG3-6-660-s002.pdf]

	defect	number of affected children	OFC at birth	OFC after age 1 yr	short stature	mental retardation	language	psychomotor delay	hyperkinesia	seizures	*facial features*	other features	MRI
**Mir et al. (2009)**	c.1423C>T, p.R475X	8		1 pt normal, 2 pts around the fifth centile, 3 pts -2/3SD	no	moderate to severe	few words	yes		pt2: yes		pt3: mild kyphosis	diminished cerebral white matter volume with sulcal enlargement, thinning of the corpus callosum, and mildly reduced cerebellar volume ; several areas of T2 hyperintensity in the subcortical white matter
**Mochida et al. (2009)**	c.1423C>T, p.R475X	3	-1,3SD; -0,3SD; -2SD	-4,1SD; -3,3SD; -5,8SD		moderate to severe	few words	no	pt1: bruxism and hand-flapping movements				thin corpus callosum and reduced volume of the cerebral white matter
**Abou Jamra et al. (2011)**	c.1423C>T, p.R475X	6		<<3rd centile	moderate to severe (15th centile to <3rd centile)	moderate to severe	absent	severe	neonatal hypotonia, stereotypic movements, hand-flapping	pt1: yes	*low frontal hairline, synophrys*	pt4,5,6: spontaneously losing their teeth and losing weight after 20y	
**Giorgio et al. (2016)**	c.1423C>T, p.R475X	1		<3rd centile	no	severe	absent	severe	stereotypic movements (hand shaking, waving, and body rocking)	no	*brachycephaly, round face, thin and horizontal eyebrows, synophrys, deep set eyes, wide nasal bridge, thin upper lip*	obesity	severe corpus callosum thinning, clear reduction of the white matter with poor myelination, normal cerebellum
**Mir et al. (2009)**	c.2311-2314 delTGTT, p.Leu772TrpfsX7	4		-1 to -3SD		severe	absent	yes		no			
**Philippe et al. (2009)**	c.1708C>T, p.R570X	3	normal	-2SD; -1SD (later -3SD); -2SD	no	severe	speech delay	yes	hyperactivity		*hypertelorism, short neck, prominent upper central incisors, short and smooth philtrum; pt2: unilateral labial cleft*	truncular obesity; pt2: long and thin fingers	unusual white matter abnormalities with discordance between T2 and FLAIR sequences (normal myelination on T2 sequence, but important white matter abnormalities on FLAIR sequence)
**Kakar et al. (2012)**	c.1024+1G>T	4		little below 3rd centile	no	severe	few words	yes	pt2: muscular weakness	no			
**Marangi et al. (2013)**	c.2851-2A4 C, p.T951Y fsX17	2		10th centile; <2nd centile	no	severe	absent	yes	hypotonia after birth; pt1: tapering fingers	pt1: yes	*peculiar craniofacial appearance, including brachycephaly, round face, hypertelorism, straight eyebrows, synophrys, wide nasal bridge, and thin upper lip with downturned mouth*	pt1: clinodactily of the fifth finger	diminished cerebral white matter volume, with marked sulcal enlargement, thinning of the corpus callosum, and reduced cerebellar volume ; several areas of T2 hyperintensity were present in the subcortical white matter
**Present patients**	c.533T>C, p.Leu178Pro	2	microcephaly at birth (OFC	-4SD; -2,8SD	no	severe	pt1: no; pt2: few words	severe	yes	pt1: yes	*pt1: sloping forehead*	no	atrophy of the corpus callosum, pt1 white matter abnormalies

			not known)										

Supplementary File 2. Clinical features in reported cases of TRAPPC9 defects and in the present probands
